# Host - Bacterial Pathogen Communication: The Wily Role of the Multidrug Efflux Pumps of the MFS Family

**DOI:** 10.3389/fmolb.2021.723274

**Published:** 2021-07-26

**Authors:** Martina Pasqua, Maria Carmela Bonaccorsi di Patti, Giulia Fanelli, Ryutaro Utsumi, Yoko Eguchi, Rita Trirocco, Gianni Prosseda, Milena Grossi, Bianca Colonna

**Affiliations:** ^1^ Department of Biology and Biotechnology “C. Darwin”, Istituto Pasteur Italia, Sapienza Università di Roma, Rome, Italy; ^2^ Department of Biochemical Sciences, Sapienza University, Rome, Italy; ^3^ The Institute of Scientific and Industrial Research (SANKEN), Osaka University, Osaka, Japan; ^4^ Department of Science and Technology on Food Safety, Kindai University, Kinokawa, Japan

**Keywords:** efflux pumps, major facilitator superfamily, bacteria host interactions, two component systems, bacterial virulence

## Abstract

Bacterial pathogens are able to survive within diverse habitats. The dynamic adaptation to the surroundings depends on their ability to sense environmental variations and to respond in an appropriate manner. This involves, among others, the activation of various cell-to-cell communication strategies. The capability of the bacterial cells to rapidly and co-ordinately set up an interplay with the host cells and/or with other bacteria facilitates their survival in the new niche. Efflux pumps are ubiquitous transmembrane transporters, able to extrude a large set of different molecules. They are strongly implicated in antibiotic resistance since they are able to efficiently expel most of the clinically relevant antibiotics from the bacterial cytoplasm. Besides antibiotic resistance, multidrug efflux pumps take part in several important processes of bacterial cell physiology, including cell to cell communication, and contribute to increase the virulence potential of several bacterial pathogens. Here, we focus on the structural and functional role of multidrug efflux pumps belonging to the Major Facilitator Superfamily (MFS), the largest family of transporters, highlighting their involvement in the colonization of host cells, in virulence and in biofilm formation. We will offer an overview on how MFS multidrug transporters contribute to bacterial survival, adaptation and pathogenicity through the export of diverse molecules. This will be done by presenting the functions of several relevant MFS multidrug efflux pumps in human life-threatening bacterial pathogens as *Staphylococcus aureus, Listeria monocytogenes, Klebsiella pneumoniae*, *Shigella/E. coli, Acinetobacter baumannii.*

## Introduction

Efflux pumps are ubiquitous transmembrane transporters able to extrude a variety of toxic compounds, including drugs from the cell (Nikaido and Pagès, 2012; Du et al., 2018; Henderson et al., 2021). The association of efflux pumps (EPs) with drug resistance was first suggested about 4 decades ago (Ball et al., 1980; McMurry et al., 1980). Since then convincing evidence has accumulated that multidrug resistance (MDR) EPs represent a major system involved in bacterial resistance to antibiotics. As compared to other antibiotic resistance systems, the expression of a single MDR EP confers the cell simultaneous resistance to multiple drugs (Nikaido and Pagès, 2012; Blair et al., 2015).

**FIGURE 1:**
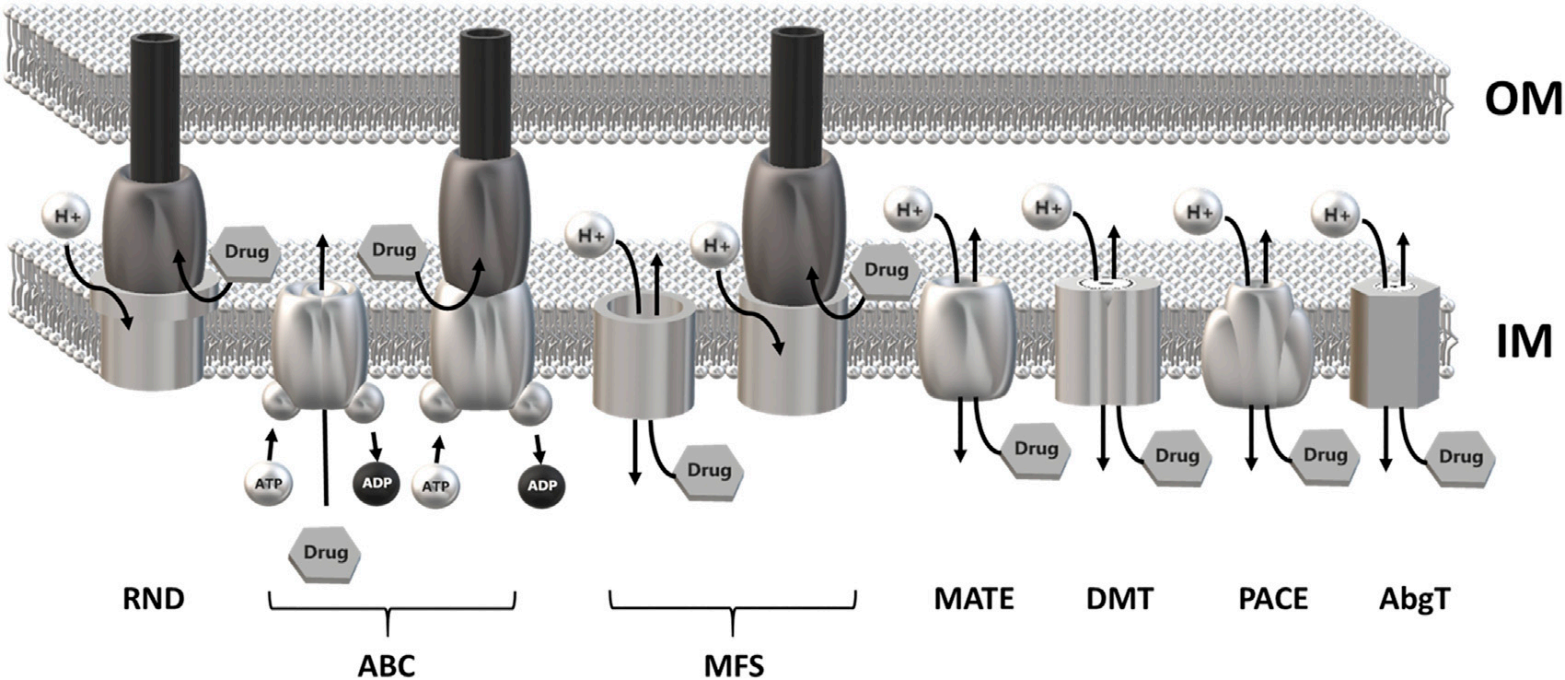
Schematic overview of the major families of multidrug efflux pumps. Most MDR EPs are single-component efflux transporter embedded within the inner membrane. Members of the RND family form tripartite efflux pumps comprising an inner membrane transporter, a periplasmatic adaptor protein and an outer membrane factor. Tripartite complexes are also present among members of the ABC and MFS superfamily. The outer membrane channel is usually constituted by TolC or a TolC-like protein. Most MDR EPs use the proton motive force of the inner membrane as energy source with the exception of the ABC superfamily which relies on ATP hydrolysis. RND, Resistance-Nodulation cell Division superfamily; ABC, ATP Binding Cassette superfamily; MFS, Major Facilitator Superfamily; MATE, Multidrug and Toxic Compound Efflux family; DMT, Drug/Metabolite Transporter superfamily; PACE, Proteobacterial Antimicrobial Compound Efflux family; AbgT, p-Aminobenzoyl-glutamate Transporter family.

MDR EPs are usually embedded in the inner membrane as a single-component transporter. In Gram-negative bacteria they can form a tripartite complex spanning the entire cell envelope. Tripartite EPs are constituted by an inner membrane transporter protein connected with an outer membrane factor (OMF) via a periplasmic adaptor protein (PAP) (Hinchliffe et al., 2013; Alav et al., 2021). MDR EPs have been identified within all major families of transporters, i.e., the Resistance Nodulation Division (RND), the ATP Binding Cassette (ABC), the Major Facilitator Superfamily (MFS), the Multidrug and Toxic Compound Extrusion (MATE), the Drug/Metabolite transporter (DMT), the Proteobacterial Antimicrobial Compound Efflux (PACE) family and the p-aminobenzoyl-glutamate Transporter (abgT) (Henderson et al., 2021). The main energy source utilized by MDR EPs is the proton motive force. Members of the ABC family rely on ATP hydrolysis (Du et al., 2018; Henderson et al., 2021) (Figure 1).

The expression of MDR EPs is strictly regulated and under usual laboratory conditions their expression is often very low (Paulsen, 2003; Sun et al., 2014; Du et al., 2018). This is not surprising since, considering their ability to transport a variety of structurally unrelated molecules, an increased expression may adversely affect the bacterial physiology. Similar EP regulatory strategies are observed in different bacteria. They are based mainly on specific TetR-, Mar- and Mer-like regulators, on global effectors as nucleoid associated proteins, or on TCS acting in response to specific environmental stimuli (Grkovic et al., 2002; Eguchi et al., 2003; Du et al., 2018; Colclough et al., 2020). Overexpression of MDR EPs has been observed in clinical isolates and has been related to the emergence of strains highly resistant to antibiotic treatment (Piddock, 2006; Beceiro et al., 2013; Li et al., 2015). Emerging evidence indicates that the physiological role of MDR EPs is not limited to antibiotic export but rather spans a wider range of functions (Alvarez-Ortega et al., 2013; Leuzzi et al., 2015; Du et al., 2018; Pasqua et al., 2019b; Kumar et al., 2020). Current data suggest that MDR EPs are relevant to bacterial pathogenicity and survival in a specific ecological niche. In this context it is worth mentioning that MDR EPs contribute to inter-bacterial communication, to biofilm formation, and to the interactions with plant and animal cells (Alcalde-Rico et al., 2016; Alav et al., 2018; Pasqua et al., 2019b). As most genes encoding MDR EPs are highly conserved and reside within the bacterial core genome, the ability of MDR EPs to facilitate antibiotic resistance is currently regarded as a fortuitous by-product of an ancient physiological function (Saier et al., 1998; Neyfakh, 2002; Teelucksingh et al., 2020; Henderson et al., 2021). This is further stressed by the fact that, based on their antibiotic extruding activity, most MDR EPs are functionally redundant and operate on the same substrate (Tal and Schuldiner, 2009; Nikaido and Pagès, 2012; Henderson et al., 2021).

It is generally assumed that MDR EPs extrude exogenous toxic molecules, including antimicrobial compounds produced by competitors. Several studies have shown that MDR EPs can also extrude endogenous metabolites and that their expression can be induced by the very metabolite they export (Henderson et al., 2021). This capacity is highly relevant to prevent intracellular accumulation of cell waste and to establish successful interactions with the host cells in order to facilitate the survival in a new niche and its colonization. Several reports highlight the involvement of MDR EPs of the MFS family in the interaction of several bacterial pathogens with their target host cells (Bigi et al., 2004; Crimmins et al., 2008; Sahu et al., 2012a; Truong-Bolduc et al., 2014; Pérez-Varela et al., 2017; Pasqua et al., 2019a; Briaud et al., 2019). MFS transporters are found in all living organisms and constitute the largest and most diverse transporter family (Law et al., 2008; Quistgaard et al., 2016). In this review we present an overview on the structural and functional role of MFS MDR EPs, highlighting their contribution in the communication with host cells, in the development of virulence and in biofilm formation. This will be done by presenting the functions of several relevant MFS multidrug efflux pumps in human life-threatening bacterial pathogens as *Listeria monocytogenes, Klebsiella pneumoniae*, *Staphylococcus aureus, Shigella/E. coli* and *Acinetobacter baumannii.*

## How Multidrug Resistance Efflux Pumps of the Major Facilitator Superfamily Export Molecules: Structure and Function

**FIGURE 2:**
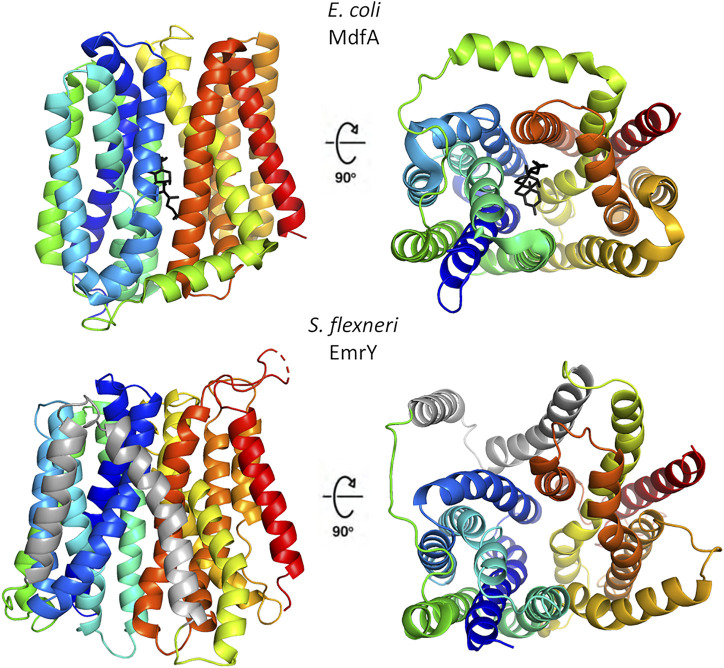
Structural organization of representative multidrug resistance MFS transporters of the DHA1 and DHA2 families. The upper panels show the 12-TM structure of *E. coli* MdfA in the inward open conformation (PDB 4zp0); in the right panel the protein is rotated by 90° to evidence the entrance to central cavity with bound chloramphenicol (black), the view is from the cytosolic side. The lower panels depict the 14-TM homology model of *S. flexneri* EmrY in the inward open conformation; the protein is shown in the same orientations of MdfA to facilitate comparison. The two additional TM helices are colored in gray. The model was obtained at the SWISS-MODEL server (Waterhouse et al., 2018) using a POT transporter (PDB 4IKV) as template. The sequence of EmrY employed as query lacks residues 434–473, which correspond to the loop connecting TM13 and TM 14 (represented as a broken line). The figure was produced with Pymol.

Despite a low degree of sequence identity, MFS transporters share a conserved structural organization, named the “MFS fold”, and a common transport mechanism that involves alternating access of the substrate binding-site to the cytoplasmic and periplasmic/extracellular side of the membrane with the transporter cycling between inward open, occluded and outward open conformations (Quistgaard et al., 2016). The MFS fold is a canonical 12 transmembrane segments (12-TM) fold consisting of two domains, known as the N- and the C-domains. Each domain is formed by 6 TM helices, connected by a cytoplasmic loop that is usually rather long and may contain an amphipathic helix (Quistgaard et al., 2016). In the N-domain, TM1, 2 and 3 are related to TM4, 5 and 6 by an approximate 180° rotation around an axis parallel to the membrane bilayer. A similar relationship is observed in the C-domain between TM7, 8, 9 and TM10, 11 and 12. In a subset of MFS transporters, belonging to the DHA2 (drug/H^+^ antiporter) and the POT (proton-dependent oligopeptide) subfamilies, this arrangement is modified by the addition of two TM segments as an insertion between the N- and C-terminal domains, giving rise to a 14-TM structure. Structural features of multidrug resistance MFS transporters are shown in Figure 2.

MFS efflux pumps involved in multidrug resistance are H^+^ antiporters of the DHA1 and DHA2 families, that couple proton translocation to drug extrusion (Du et al., 2018). DHA1 members generally exhibit 1:1 drug:H^+^ stoichiometries whereas transporters belonging to the DHA2 family exchange two protons with one substrate. While 3D structures have been determined for DHA1 transporters in different conformational states (*Escherichia coli* MdfA represents the prototypical EP of this group), no structure is available for DHA2 family members yet. Detailed structural studies of MdfA have allowed to gain in-depth information on the substrate-binding site and on the molecular mechanism of proton-coupled drug transport (Heng et al., 2015; Nagarathinam et al., 2018; Wu et al., 2020). In MFS transporters the substrate-binding site is generally found in a central cavity, with residues from both the N- and C-terminal domains contributing to ligand recognition. Polyspecificity of MDR transporters is achieved by means of a large substrate-binding pocket, which is able to accommodate compounds with different sizes and shapes. Further flexibility in multidrug recognition has been suggested to be conferred by the very low number of H-bonds established with the substrates. In MdfA substrate binding is mediated mainly through hydrophobic, van der Waals and polar contacts, which are geometrically much less stringent than H-bonds and likely allow structurally and chemically diverse drugs to adopt different orientations within the large central cavity (Wu et al., 2020). Conserved acidic residues E26 and D34 in motif-D located in TM1 are implicated in proton and substrate transport coupling, and it has been proposed that substrate-binding dependent changes in their protonation state trigger conformational changes required for transport (Heng et al., 2015). Recently, a model of the 14-TM MFS QacA, a DHA2 protein with a broad substrate repertoire that confers MDR to *S. aureus,* has been proposed (Majumder et al., 2019). The additional TM helices TM7 and 8, which form a V-shaped insertion between the N- and C-domains, have been demonstrated to be required for the function of the transporter in resistance to different toxic compounds. Moreover, the critical role of specific (de)protonatable acidic residues in TM1 and TM13 for the recognition and transport of cationic antibacterial compounds was revealed (Majumder et al., 2019). Structure-based sequence analyses indicate that many other multidrug MFS EPs share hydrophobic and large substrate-binding sites, in line with their ability to extrude lipophilic, cationic and neutral substrates.

The transport mechanism of MFS transporters has been described initially as a “rocker-switch” and then revised as a “clamp-and-switch” model in which the transition between the different states involves not only rigid-body rotations, but also structural changes in individual transmembrane helices (Quistgaard et al., 2016). In particular, kinking and twisting of TM5 in the outward open conformation of MdfA appears to be crucial for the transition from the inward open to the outward open state, together with reorganization of part of the hydrophobic core of the N-terminal domain and of a cytoplasmic loop of the C-terminal domain to close the cytoplasmic entrance (Nagarathinam et al., 2018). It is worth to point out that TM5 hosts motif-C (gxxxGPxxGGxl), a conserved sequence defined as the “antiporter motif”, where substitution of the proline residue leads to loss of function of MdfA (Heng et al., 2015). The MFS signature motif-A (GxLaDrxGrkxxxl) is located between TM2 and TM3 and plays a role in the stabilization of the outward open state (Henderson et al., 2021). Interestingly, motif-A appears not to be fully conserved in a set of DHA2 transporters comprising EmrY from *S. flexneri*, Tet38 from *S. aureus* and MdrM and MdrT from *L. monocytogenes* where the sequence deviates from the canonical arrangement (M.C. Bonaccorsi di Patti, unpublished observation), suggesting an unexpected flexibility in the mechanisms of these transporters. Other sequence motifs have been identified in MFS transporters of the DHA2 family, and are partly conserved in the MDR EPs cited above, including motif-D1 (lDxTvxnAlP) in TM1, with a probable role in proton-coupling mediated by the aspartate residue as in DHA1 transporters, and motif-H (WxwxFlINvPig) in TM6 with as-yet uncertain function (Henderson et al., 2021). A striking feature of MdfA, possibly shared by other MDR MFS transporters of DHA1 and DHA2 families, is a high degree of flexibility in the architecture of the substrate and H^+^ binding sites whereby the protein can accommodate multiple substitutions without significant loss of drug:H^+^ coupling (Wu et al., 2020). This finding might provide an explanation to the observation that in MDR EPs the typical sequence motifs described in MFSs appear to tolerate more variation in sequence (and possibly position) than expected.

Most MFS EPs act as single monomeric units. However, a limited number of tripartite systems have been identified which span the entire cell envelope in Gram-negative bacteria (Figure 1). In the latter case, the MFS component is connected to a periplasmic adaptor protein, which in turn is linked to TolC or to a TolC-like protein in the outer membrane to provide a continuous sealed channel for translocation of the substrate across the cell envelope (Alav et al., 2021). At variance with tripartite assemblies involving ABC and RND transporters, tripartite assemblies including MFSs are still poorly characterized from a structural point of view. Experimental 3D structures are available for single components belonging to different assemblies, such as the adaptor protein EmrA from *Aquifex aeolicus* (Hinchliffe et al., 2014) and the TolC-like outer membrane trimeric protein VceC from *Vibrio cholerae* (Federici et al., 2005). The MFS component of the system belongs to the 14-TM DHA2 family and it has been proposed that it may function as a dimer (Symmons et al., 2015). It is tempting to speculate that the two additional TMs typical of DHA2 transporters could contribute to dimerization of the MFS. Tripartite systems discussed in this review are represented by *S. flexneri* EmrKY and *K. pneumoniae* KpnGH. In both cases the outer membrane component is not encoded in the same operon as the periplasmic adaptor protein and the EP, and it is constituted by TolC. The periplasmic adaptor proteins EmrK and KpnG share homology to *E. coli* EmrA and by analogy they are predicted to be hexameric in the assembly, providing the molecular link between the EP in the inner membrane and TolC in the outer membrane. EmrY and KpnH are predicted to present a more than 40 amino acid-long extracellular loop between TM13 and TM14, which might be involved in interactions with EmrK and KpnG, respectively. However, this hypothesis should be viewed with caution because a similar long loop is present also in the single monomeric EP QacA of *S. aureus*.

## *Listeria monocytogenes:* The Efflux Pumps MdrM and MdrT Play a Crucial Role in the Activation of the Host Cytosolic Surveillance Pathway

*L. monocytogenes* is a Gram-positive food pathogen responsible for high mortality in newborns as well as in pregnant women and in immunocompromised individuals. After invading phagocytic and non phagocytic cells, *L. monocytogenes* escapes the initial vacuole and spreads to adjacent cells by recruiting host actin filaments. In response to growing cytosolic bacteria host cells induce a type I interferon innate immune response. In particular, there is an enhanced expression and secretion of IFN-β (Pizarro-Cerdá and Cossart, 2018). An essential step in the induction of IFN-β is the release of cyclic-di-AMP (c-di-AMP) by *L. monocytogenes* (Woodward et al., 2010; Kaplan Zeevi et al., 2013). Several studies have shown that this molecule serves as an important second messenger influencing key processes in bacteria, such as sporulation, response to cell wall stress, peptidoglycan homeostasis, and genome surveillance (Yin et al., 2020). In bacteria, c-di-AMP is synthetized by diadenylate cyclase (DAC), using ATP as a substrate. Conversely, c-di-AMP is linearized to 5′-pApA by a specific phosphodiesterase (PDE) (Yin et al., 2020). Recent observations on the effect of c-di-AMP on intercellular communication highlight its relevance to the virulence of *L. monocytogenes*. In hepatocytes, a major reservoir of *L. monocytogenes* in systemic infections, it has been shown that c-di-AMP is recognized by the oxidoreductase RECON (Reductase Controlling NF-κB). This interaction determines the inhibition of RECON’s activity as repressor of NF-κB (McFarland et al., 2017). Augmented NF-kB activation increases the expression of nitric oxide synthase and, in turn, higher levels of nitric oxide favor spreading of *L. monocytogenes* in a variety of host cells (McFarland et al., 2018).

The role of MFS transporters in the interplay of *Listeria monocytogenes* with host cells was first evidenced in a wide genetic screening of mutants inducing altered host innate immune response in infected macrophages (Crimmins et al., 2008). Two EPs of the MFS superfamily, MdrM and MdrT, turned out to be involved in the cytosolic immune response in infected host cells. The MdrM and MdrT EPs are closely related to the well-studied MDR transporter QacA of *S. aureus* (Crimmins et al., 2008). They are expressed at low level and each is negatively controlled by a specific repressor (MarR and BtrA, respectively), located upstream of the EP gene (Crimmins et al., 2008; Quillin et al., 2011). *L. monocytogenes* strains defective in the *mdrM* or *mdrT* genes exhibit a significant reduction of several cytokines, including interferon-β (IFN-β), produced by infected host cells following infection (Crimmins et al., 2008; Woodward et al., 2010; Kaplan Zeevi et al., 2013). On the other hand, silencing of the cognate repressors MarR and BrtA has the opposite effect, triggering a strong host immune response. LC-MS analyses reveal a high level of c-di-AMP in supernatants of *L. monocytogenes* strains overexpressing MdrM and MdrT, suggesting that during intracellular growth of the bacterium these transporters favor the export of c-di-AMP (Woodward et al., 2010).

MDR transporters are often functionally redundant due to their overlapping substrate specificity. Genome analyses have shown that *L. monocytogenes* carries, besides *mdrT*, other genes (*mdrA* and *mdrC*) homologous to *mdrM.* These genes, collectively defined as MTAC transporters, are transcriptionally upregulated during intracellular growth and contribute to the type I interferon response by host cells (Kaplan Zeevi et al., 2013). While MTAC transporters are not required for intracellular growth in macrophages, infections in mice reveal that these EPs are active and contribute to bacterial virulence. Indeed, in infections with a DMTAC mutant a lower number of bacteria is found in livers and spleens of infected mice (Kaplan Zeevi et al., 2013). Besides triggering IFN-β production during bacterial infection, MTAC transporters play a role in the response to cell wall stress. In the presence of a sublethal concentration of vancomycin, *L. monocytogenes* DMTAC mutants are unable to increase peptidoglycan production, a drug titration mechanism normally employed by the bacterium. The involvement of MTAC transporters and c-di-AMP in the control of cell wall maintenance during stress conditions has been further demonstrated by altering the level of c-di-AMP. In a *L. monocytogenes* DMTAC mutant the increased expression of c-di AMP cyclase reduces the susceptibility to gentamycin, while over expression of c-di-AMP phosphodiesterase increases the sensitivity to the drug. MTAC transporters are also required for the synthesis of LTA under normal growth conditions, likely via c-di-AMP efflux. These data, together with the fact that mutants defective in LTA synthesis enhance the MdrM-dependent type I interferon response, lead to the hypothesis that MTAC transporters are involved in the management of LTA stress, and more generally in cell wall maintenance and in the interplay with host cells (Kaplan Zeevi et al., 2013).

The contribution of MdrM and MdrT is not limited to promoting the host innate immune response. These MFS EPs affect *L. monocytogenes* survival in the bile-rich niches the bacterium encounters during infection, i.e. liver, spleen and gallbladder. In particular, both *mdrT* and *mdrM* genes are strongly induced by cholic acid, an essential bile component and their deletion significantly attenuates bacterial virulence *in vivo* in livers of infected mice and *in vitro* in the presence of bile (Quillin et al., 2011). High induction of *mdrT* depends on the loss of the ability of the BrtA repressor to bind and repress the *mdrT* promoter in the presence of cholic acid. It is yet unclear whether this depends on cholic acid directly binding to the BtrA protein or perturbing the structure of BtrA because of its detergent properties.

## *Staphylococcus aureus:* The Efflux Pump Tet38 as Key Element in the Interaction With Host Cell

*S. aureus* is a versatile pathogen, able to cause acute and chronic infections in animals and humans. Its arsenal of virulence factors and its large panel of MDR EPs enable it to survive within the host cell environment by resisting antibiotic therapy and escaping host defences. While *S. aureus* is not an obligate intracellular pathogen, it can invade and survive within non professional phagocytic cells such as epithelial and endothelial cells. After internalization and fusion with the lysosomes, *S. aureus* can replicate rapidly and then escape from the phagolysome (Garzoni et al., 2007). The Tet38 EP plays an essential role in the virulence of *S. aureus*, actively contributing to the invasion and survival of the bacteria within epithelial cells.

Tet38 is a single-peptide, chromosomally encoded MFS efflux pump, highly conserved among *S. aureus* strains. It confers resistance to several compounds, including tetracyclin, fosfomycin, glycerol-3-phosphate and some unsaturated fatty acids as palmitoleic and undecanoic acid (Truong-Bolduc et al., 2014). The Tet38 protein (48 KDa) has 14 predicted TM segments. Except for the positions of glutamate residues, the organization of the TM segments is similar to that observed in *S. aureus* TetK and *B. subtilis* TetL EPs, two MFS efflux pumps involved in the extrusion of tetracycline. The expression of *tet38* is tightly regulated and is sensitive to stimuli from the host and from the outer environment. Two factors, MgrA and TetR21, negatively control *tet38* expression. While TetR21 represses *tet38* by binding specifically to its promoter, MgrA acts indirectly by binding the *tetR21* promoter (Truong-Bolduc et al., 2005; Truong-Bolduc et al., 2015).

Evidence on the involvement of Tet38 in *Staphylococcus* pathogenesis came initially from the observation that the expression of *tet38* and *norB* (another MFS EP) was selectively increased in abscesses (Ding et al., 2008) and that Tet38 contributed significantly to bacterial survival and replication in the abscess environment (Ding et al., 2008). In particular, the strong induction of Tet38 correlated with a high presence in abscesses of typical Tet38 fatty acid substrates as palmitoleic and undecanoid acid (Truong-Bolduc et al., 2014). Successive observations have revealed that colonization of the skin surface in a mouse model was strongly reduced in *S. aureus tet38* mutants as compared to wt strains, and that the wt level was restored by ectopic expression of a functional Tet38 pump (Truong-Bolduc et al., 2014).

Tet38 affects several steps of the invasion process of *S. aureus,* including adhesion, internalization and trafficking in epithelial cells. Invasion assays of epithelial and endothelial cells have shown that Tet38 plays an essential role in the initial step of the internalization of *S. aureus* in host cells. Indeed, the absence of Tet38 strongly affects bacterial uptake (Truong-Bolduc et al., 2015). Using antibodies against the scavenger receptor CD36, it has been observed that the CD36 blockade reduced internalization of the wild type *S. aureu*s, while it had no effects on *tet38* deletion mutants (Truong-Bolduc et al., 2017). The dependence of internalization on CD36 of wild type *S. aureus* suggested a direct interaction between Tet38 and CD36. This interaction has been confirmed by column-retention assays with purified Tet38 and CD36 (Truong-Bolduc et al., 2019) and the Tet38 amino acid residues essential for *S. aureus* internalization have been recently identified (Truong-Bolduc et al., 2021). Truong-Bolduc et al. (2019) also demonstrated that Tet38 can bind *in vitro* CD36 in the heterodimeric CD36-TLR2 complex and form the heterotrimeric Tet38-CD36-TLR2 complex, suggesting that Tet38 might influence the sensitivity of TLR-2 to the presence of lipoteichoic acid (LTA), a bacterial component that actively participates to host cell invasion. Due to its localization within the inner membrane, Tet38 is surrounded by layers of peptidoglycan, LTA and teichoic acid (WTA). These authors also indicate that Tet38 hampers the activity of tunicamycin, an inhibitor of WTA synthesis. Indeed, resistance to tunicamycin increases with the upregulation of the *tet38* gene while strains lacking Tet38 become fully sensitive to the drug. A similar behaviour has been reported with Congo Red (CR), an inhibitor of LTA synthesis. The involvement of Tet38 in these resistance phenotypes was further confirmed by observing that sensitivity to tunicamycin and CR is restored in the presence of the EP inhibitor reserpine.

The Tet38 EP also contributes to the subsequent step of the *S. aureus* infection in epithelial cells, in terms of both bacterial viability and trafficking in phagolysosomes. As compared to *tet38* mutants, after the fusion of *S. aureus*-associated endosomes with lysosomes, the viability of wt cells is higher and a smaller number of bacteria is associated with phagolysosomes. Moreover, Tet38 favours the escape from the phagolysosome. Indeed, buffering the acidic phagolysosome environment with chloroquine rapidly increases both viability and lysosome escape of wt *S. aureus* while it has no significant effect on *tet38* mutants (Truong-Bolduc et al., 2017).

Recently, it has been observed that during coinfection with *S. aureus* and *Pseudomonas aeruginosa* the expression of *S. aureus* Tet38 is strongly increased (Briaud et al., 2019). These bacteria occupy the same anatomical niche during airway infections in cystic fibrosis patients. In the initial phase of the infection, *P. aeruginosa* outcompetes *S. aureus* by producing specific growth inhibitory compounds. As the infection progresses towards a chronic state, the interaction between the two species evolves into coexistence. Transcriptomic studies using pairs of strains of both species reveal that the presence of *P. aeruginosa* induces a dysregulation of the *S. aureus* expression profile. In particular, during coexistence the predominant transcriptomic alteration is the upregulation of *S. aureus tet38*, caused by the reduced expression of the Tet38 MgrA repressor gene. This alteration leads to an increased antibiotic tolerance and to a higher uptake rate of *S. aureus* into epithelial pulmonary cells, two essential determinants of chronic infection (Briaud et al., 2019). Altogether, the evidence currently available indicates that the Tet38 EP is a potential target for new antibacterial strategies able to block the internalization of *S. aureus*, a crucial step in the recurrence and persistence of *S. aureus* infections.

## The Efflux Pump Emrky of *Shigella flexneri*: The Crucial Role of Two Component Systems in Sensing and Responding to the Host Environment

*Shigella* is an intracellular pathogen that can invade and colonize the intestinal epithelium, resulting in severe inflammatory colitis (The et al., 2016; Pasqua et al., 2017). After being ingested by the host, *Shigella* reaches the colon and rectum, where it is endocytosed by M cells in the Peyer’s patches. The bacteria are then released into the intraepithelial pocket, where they invade resident macrophages. Once inside macrophages, *Shigella* disrupts the phagocytic membrane and multiplies within the cytosol. Then it induces macrophage death via pyroptosis. After being released by the dead macrophages, *Shigella* invades the intestinal epithelial cells, where it multiplies and disseminates in cells (Ashida et al., 2011). The ability to survive and replicate inside macrophages and intestinal epithelial cells is regarded as a key virulence feature of the bacterium.

In a recent attempt to investigate on the possible role of *Shigella* EPs in pathogen-host interactions, MDR EP encoding genes of *S. flexneri* M90T and *E. coli* K-12 were compared (Pasqua et al., 2019a). Only 14 out of 20 EP-encoding genes in *E. coli* were conserved in *S. flexneri*. Two MFS EPs (*mdtG* and *mdtM*) and one RND EP (*acrEF*) were absent from the *S. flexneri* genome, whereas three RND EPs (*cusB*, *mdtABC*, and *mdtEF*) were not functional due to gene disruption. Silencing of several EP encoding genes in *S. flexneri* is not surprising as in *Shigella* many housekeeping genes present in the commensal *E. coli* ancestor have been lost following a pathoadaptation process (Prosseda et al., 2012; Leuzzi et al., 2017). Following an extensive analysis of the expression of the 14 EPs of *S. flexneri* M90T, carried out in *Shigella-*infected human U937 monoblasts after differentiation into macrophage-like cells and in *Shigella*-infected Caco-2 epithelial cells, the transcriptional profiles were found to vary across EPs and between macrophages and epithelial cells. In particular, in macrophages expression of *emrA*, *emrD*, *emrE*, *emrK*, and *mdtJ* increased whereas that of *acrA* decreased. In Caco-2 cells *acrD*, *emrD*, *emrE*, *macA*, *mdtA*, *mdtJ*, *ydhE*, and *yidY* were overexpressed while *acrA*, *emrA*, and *emrK* showed a reduced level of expression (Pasqua et al., 2019a). Considering that under ordinary laboratory growth conditions *E. coli* constitutively expresses *acrAB* (and *tolC*) while most other EP-encoding genes are repressed (Sulavik et al., 2001), the decreased expression of *Shigella acrA* in macrophages and epithelial cells suggests that in the functioning MDR EP pool a shift from AcrAB to other pumps may have occurred. Interestingly, the *Shigella emrK* gene, which belongs to the *emrKY* operon, is strongly overexpressed in macrophages but is downregulated in Caco-2 epithelial cells (Pasqua et al., 2019a). In *E. coli emrKY* expression is activated in response to the coordinated action of a mild acidic environment and a high concentration of alkali metals, a combination which is sensed by the EvgA/EvgS TCS (Eguchi and Utsumi, 2014). Upregulation of *Shigella emrK* in macrophages can be likely assumed to depend on the same stimuli. Indeed, the uptake of *Shigella* by macrophages triggers a decrease of the intracellular pH favoring the activation of the *emrKY* by the EvgA regulator. Further analyses have revealed that deleting *emrKY* from *S. flexneri* M90T leads to a reduced growth rate in minimal medium (pH 6.0) supplemented with 0.2 M KCl, and to an intracellular growth disadvantage of the mutant in competition assays with the parental strain. This strongly suggests that EmrKY contributes to the fitness of *Shigella* in macrophages (Pasqua et al., 2019a).

**FIGURE 3:**
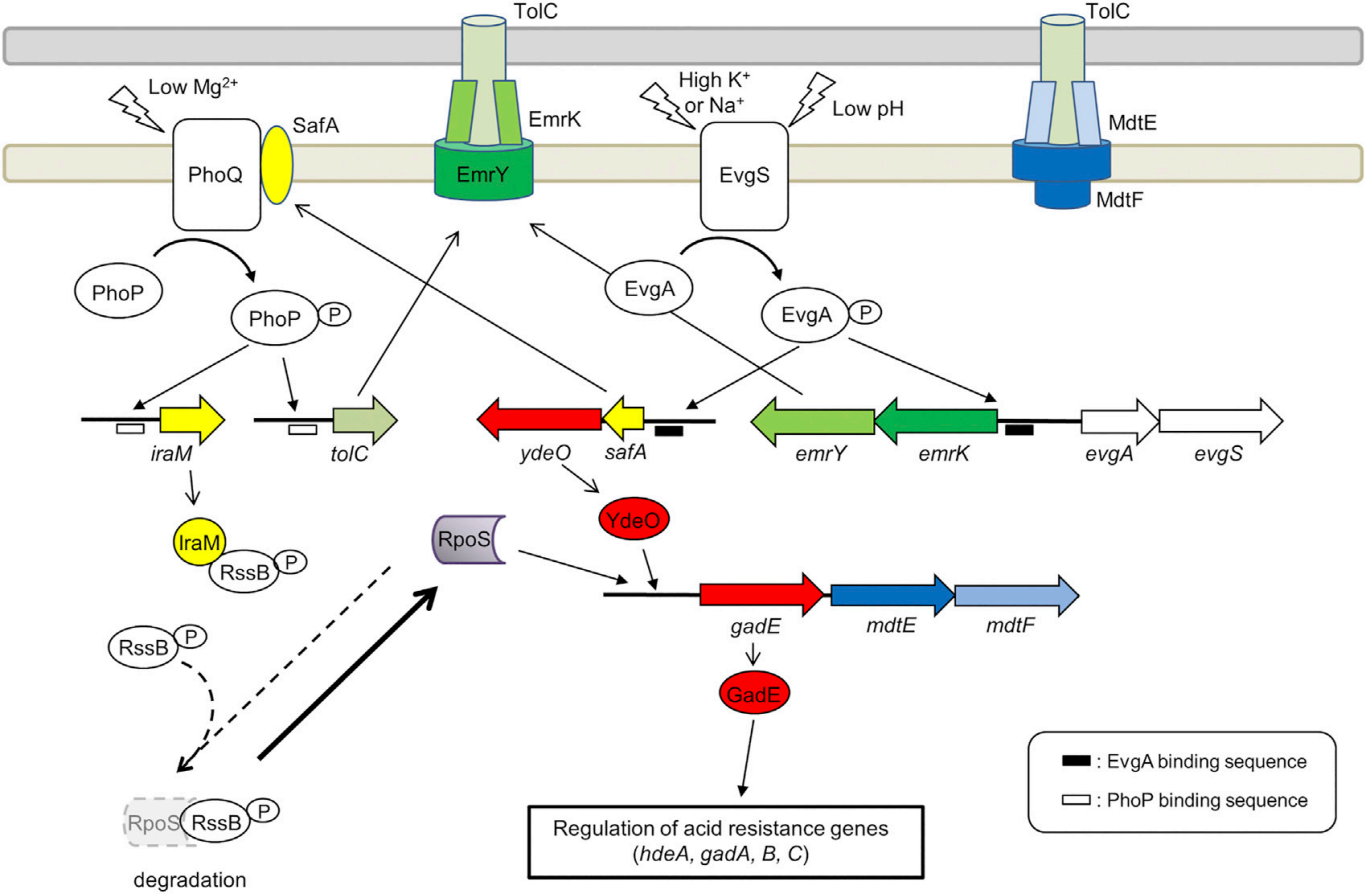
Role of the EvgA/EvgS two component system in the activation of MDR EP encoding genes *emrKY* and *mdtEF*. The figure shows the EvgA-YdeO-GadE cascade and the SafA-PhoQ/PhoP-IraM-RpoS network in *E. coli.* The genes are highly conserved between *S. flexneri* M90T and *E. coli* MG1655 (the amino acid sequence homology is 98–100%), except *iraM*, *mdtE,* and *mdtF*. The *iraM* gene shows 61.7% amino acid homology in *S. flexneri* M90T, but has 93.5% amino acid homology in *S. sonnei*. Although *mdtE* and *mdtF* are disrupted in *S. flexneri*, they are conserved in *S. dysenteriae*, *S. boydii*, and *S. sonnei*. EvgA-P, PhoP-P, YdeO, RpoS, and GadE positively regulate the target genes (indicated by arrows). EvgA-P and PhoP-P also induce the expression of *gadE-mdtEF* by directly binding to the *gadE* promoter.

EmrKY is a member of the MFS EP family. This pump was originally identified in *E. coli* by analyzing genes adjacent to the *evgAS* operon, which encodes the EvgS/EvgA TCS (Tanabe et al., 1997) (Figure 3). EmrKY functions as a tripartite EP with TolC as an outer membrane factor (Eguchi et al., 2003). Induction of *emrKY* expression in *E. coli* conferred resistance to deoxycholate but not to cholate, SDS, and several antimicrobial agents and dyes (Kato et al., 2000; Nishino and Yamaguchi, 2002). More recently, it has been reported that overexpression of *tolC* and *emrKY* enhanced amorphadiene transport, a precursor of the antimalarial drug artemisinin (Zhang et al., 2016). However, to the best of our knowledge, there is no additional information available about the substrates that are pumped out by EmrKY. *E. coli* cells with mutations in *emrK* and *emrY* are sensitive to mitomycin C, UV irradiation, and H_2_O_2_, which suggests that EmrKY eliminates toxic metabolites induced by DNA damage (Han et al., 2010). This may help understanding how EmrKY contributes to the survival of *Shigella* in macrophages. No data on the role of EmrKY in the virulence of other *Enterobacteriaceae* have been reported so far.

The expression of *emrKY* in *E. coli* is induced by the EvgS/EvgA TCS, where EvgA, the response regulator, directly binds to the promoter region of *emrKY* (Kato et al., 2000). In addition to *emrKY*, EvgS/EvgA upregulates a network of acid resistance genes, through a cascade of EvgA-YdeO-GadE regulators (Masuda and Church, 2003; Itou et al., 2009). It positively controls also the SafA-PhoQ/PhoP-IraM-RpoS network, which is composed of two TCSs, two TCS-connecting small proteins, and a sigma factor (Eguchi et al., 2007; Eguchi et al., 2011) (Figure 3). The small membrane protein SafA directly binds and activates PhoQ at the inner membrane (Eguchi et al., 2012; Yoshitani et al., 2019) and the anti-adapter protein IraM, which is induced by activated PhoQ, binds to the response regulator RssB preventing it from interacting with RpoS, the general stress responding sigma factor (Bougdour et al., 2008). This protects RpoS from proteolysis by ClpXP and results in the accumulation of cellular RpoS during the exponential phase. Both PhoP and RpoS contribute to the upregulation of *gadE*, the major regulator of acid resistance genes. The upregulation of these genes confers severe acid resistance to exponentially growing *E. coli* cells (Itou et al., 2009). Notably, *tolC* is also upregulated by EvgS/EvgA via SafA and PhoQ/PhoP (Figure 3). EvgS/EvgA enhances the expression of both *emrKY* and *tolC*, contributing to the formation of EmrKY-TolC tripartite EP (Eguchi et al., 2003). Interestingly, all genes involved in the EvgA-YdeO-GadE cascade and in the SafA-PhoQ/PhoP- IraM-RpoS network are conserved in *Shigella*, suggesting that these systems may favor, among other processes, the expression of EPs relevant to the survival of *Shigella* in macrophages. Indeed, using a *Shigella* strain carrying a *emrK-gfp* fusion, it has been shown that in macrophages the fusion is not expressed in a *evgA* background, indicating that during infection the expression of *emrK* is induced by the activation of the EvgS/EvgA TCS (Pasqua et al., 2019a).

The crucial role of the EvgA/EvgS TCS in activating the EPs required for a successful interaction with host cells is further highlighted by the analysis of the expression profile of MDR EPs in adherent-invasive *Escherichia coli* (AIEC) (Fanelli et al., 2020). Similarly to *Shigella, emrK* expression in LF82, a strain isolated from Crohn disease lesions, is induced inside macrophages. However, unlike *Shigella, emrK* expression occurs also in AIEC-infected Caco-2 cells*.* The expression of *mdtEF*, which encodes a RND EP, is induced in AIEC cells inside macrophages but not inside epithelial cells. MdtEF is known to contribute to the survival of AIECs inside macrophages (Fanelli et al., 2020). As shown in Figure 3, *mdtEF* is co-transcribed with *gadE* as a *gadE-mdtEF* operon. EvgS/EvgA is one of the many systems that control the expression of this operon (Arcari et al., 2020) (Figure 3). It is assumed that the environment inside macrophages is acidic and oxidative, hosts toxic molecules such as antimicrobial peptides, causes genotoxic stress, and lacks important nutrients (Demarre et al., 2019). The acidic conditions and the lack of nutrients may serve as activating signals for the EvgS sensor. Indeed, deletion of *evgAS* significantly affects the ability of AIECs to survive and multiply within macrophages (Demarre et al., 2019), indicating that EvgS/EvgA is activated in macrophages. It should be noted that although *mdtEF* is disrupted in *S. flexneri*, the *gadE-mdtEF* operon is conserved in *Shigella dysenteriae*, *Shigella boydii*, and *Shigella sonnei* (BioCyc database collection, https://www.biocyc.org/). As shown in Figure 3, *mdtEF* and *tolC* expression regulated by the EvgA-YdeO-GadE cascade and the SafA-PhoQ/PhoP- IraM-RpoS network may contribute to the survival of these *Shigella* subspecies in macrophages.

Additional signals for activation of EvgS have been reported. Indole inhibits EvgS at concentrations that are typically found in the intestine (Boon et al., 2020). Anaerobic conditions also inhibit EvgS activity, and it has been proposed that EvgS activation requires oxidation in addition to mild acidic conditions (Inada et al., 2021). The current model suggests that EvgS/EvgA is silenced in the intestine due to the presence of indole and anaerobic conditions. EvgS/EvgA is activated after *Shigella* or AIEC invade macrophages, and thereafter induces the expression of EmrKY and other EPs. EvgS/EvgA is widely conserved within the *E. coli* species. Some reports have associated EvgS/EvgA to the pathogenicity of *E. coli* other than AIEC and *Shigella*. In the case of enteropathogenic *E. coli*, EvgA represses the expression of the type III secretion system responsible for delivering a set of effector proteins into the host cell cytoplasm (Nadler et al., 2006). In avian pathogenic *E. coli* the silencing of *evgS* attenuates lung colonization (Dziva et al., 2013). Moreover, a recent study has shown that EvgS/EvgA activation protects *E. coli* from gallium nitrate induced death. It has been suggested that EvgS/EvgA upregulated genes encode enzymes involved in ROS detoxification and in the glyoxylate shunt of the TCA cycle (Zeng et al., 2021). These results may provide clues to improve our understanding of how EmrKY contributes to survival within macrophages.

## Building Biofilms in *Acinetobacter baumannii*: The Contribution of Major Facilitator Superfamily Efflux Pumps in Exporting Biofilm Key Molecules

In order to survive hostile conditions many microorganisms, including pathogens, are able to organize their communities as biofilms in response to different environmental stimuli (e.g., nutrient and metabolic signals, host-derived molecules and antibiotics) (Flemming et al., 2016). The biogenesis of a biofilm is a complex process that requires intense cell-to-cell communication through quorum sensing signals and is usually articulated into major steps: attachment of planktonic cells to living substrates or to abiotic surfaces (e.g., medical devices, domestic or industrial environments); cell proliferation, resulting in the formation of microcolonies; and eventually growth and maturation into a fully differentiated biofilm with self-production of an extracellular matrix, composed of lipids, nucleic acids, polysaccharides and proteins, that ensures the structural integrity of the film and its adhesion to surfaces (Karatan and Watnick, 2009; Flemming and Wingender, 2010). Bacteria embedded in a mature biofilm can further spread and colonize new surfaces through dispersion of individual cells (Flemming et al., 2016).

The ability to form biofilms is considered a major virulence factor in many bacterial human pathogens. *A. baumannii* represents an extensively studied microorganism whose interaction with the host is mainly based on biofilm formation (Harding et al., 2018). In clinical environments *A. baumannii* is considered a serious threat because of its so-called “persist and resist” strategy (Harding et al., 2018). It depends on the combination of two factors: a remarkable ability to persist under unfavorable conditions as dormant cells or biofilms and a marked propensity to develop multiple antibiotic resistances (Harding et al., 2018). This strategy, together with the expression of specific virulence factors, allows *A. baumannii* to establish severe infections characterized by high mortality, e.g., bloodstream and catheter-associated urinary infections, ventilator-associated pneumonia, and skin and soft tissue infections (Harding et al., 2018). Several EPs of the MFS and RND families contribute to the ability of *A. baumannii* to form biofilms and develop multi-drug resistant phenotypes (Alav et al., 2018; Pasqua et al., 2019b).

The first evidence on the involvement of an MFS EP in the biosynthesis of *A. baumannii* biofilm came from the analysis of the *pmt* gene in the highly multidrug resistant pathogenic strain AIIMS 7 (Sahu et al., 2012a). The *pmt* gene encodes a 17 KDa inner membrane MFS transporter which is highly expressed during adherence to a substrate. In particular, upregulation of *pmt* occurs mainly during the initial stages (24–48 h) of biofilm formation. The Pmt protein drives the adherence of the bacterium onto both biotic and abiotic surfaces, making the biofilm dense and thick. The capability of Pmt to confer adherence onto host cells was confirmed by the observation that ectopic expression of *pmt* in *E. coli* strongly increases its adherence onto HeLa and *S. cerevisiae* cells (Sahu et al., 2012a).

Bacterial cell aggregates and biofilms are structures known to be encased in an extracellular matrix composed by various macromolecules, including nucleic acids, which serve as scaffolding agents to stabilize the biofilm structure especially in the initial phases of its formation (Whitchurch et al., 2002; Flemming and Wingender, 2010). In *A. baumannii* it has been shown that extracellular DNA (eDNA) is actively released during early growth phase and plays a crucial role in bacterial aggregation, forming a network-like structure (Sahu et al., 2012b). The Pmt EP is surmised of being responsible for the export of nucleic acids as it is expressed in early stages of biofilm formation (Sahu et al., 2012a). This hypothesis is also supported by the observation that in *E. coli* strains harboring the *A. baumannii pmt* gene there is an increased release of eDNA (Sahu et al., 2012b). Besides Pmt, another MFS efflux pump, AbaF, is involved in the formation of *A. baumannii* biofilms. The capacity of AbaF to extrude fosfomycin (Sharma et al., 2017) makes the cell resistant to this drug. Experiments performed with an *abaF* defective *A. baumannii* strain show that, in addition to being sensitive to fosfomycin, the mutant is also sensitive to chloramphenicol and is impaired in biofilm development. In most clinical *A. baumannii* strains the *abaF* gene is constitutively expressed. This is thought to depend not only on the capacity of AbaF to pump out antibiotics but also on its involvement in the extrusion of biofilm material (Sharma et al., 2017). The observation that *Caenorhabditis elegans* fed on a *A. baumannii abaF* mutant shows a higher survival rate as compared to feeding on the wt bacterium strongly suggests that AbaF contributes to bacterial virulence, likely by pumping out host derived antibacterial factors (Sharma et al., 2017).

Biofilm formation is essentially the progressive embedding of a bacterial community in an extracellular matrix (Flemming and Wingender, 2010). In this process, the ability of the cells to move and colonize the environment where they reside is crucial. Indeed, motility is considered as a key factor in the infectiveness of pathogens (Pérez-Varela et al., 2017). In *A. baumannii* the AbaQ MFS transporter, initially characterized for conferring resistance to quinolones (Pérez-Varela et al., 2017), is widely present in clinical isolates (Pérez-Varela et al., 2018). It is a 47.8 kDa protein encoded by a 1305 bp gene and is composed of 12 TM segments. In a nematode model of *A. baumannii* virulence, the inactivation of the *abaQ* gene causes the loss of surface-mediated motility and the attenuation of virulence (Pérez-Varela et al., 2017). Surface-associated motility is a crucial requirement for biofilm formation in *A. baumannii* as the bacterium lacks flagella. The association between motility and MFS EPs has been observed also in *Pseudomonas putida* by studying the role of the MFS EP PcaK in the chemiotaxis towards 4-hydroxybenzoate (4-HBA), which is the pump substrate (Nichols and Harwood, 1997). The involvement of EPs in surface-associated motility and virulence in *A. baumannii* is not limited to MFS transporters. Recent evidence indicates that EPs belonging to other superfamilies contribute to the motility of the cells and to full expression of the virulence phenotype (Pérez-Varela et al., 2019). Altogether, these data further support the relevance of MFS EPs not only to antibiotic resistance but also to specific steps of biofilm formation, cellular movement and virulence in an emergent pathogen as *A. baumannii.*

## *Klebsiella pneumoniae:* KpnGH and the Response to Gastro-Intestinal Stress

*K. pneumoniae* is an opportunistic pathogen and a leading cause of severe illness in nosocomial patients (Wyres et al., 2020). Indeed, the significant incidence (5–7%), of hospitalized and immunocompromised patients affected by pneumonia, septicaemia, urinary tract and soft tissue infections caused by this bacterium, makes it a paradigm of community-acquired infections worldwide. The high mortality rate is further increased by the emergence of multidrug resistance phenotypes, ultimately resulting in failure of drug therapies. Most genes conferring resistance to clinically relevant antibiotics are located on large conjugative plasmids and have been acquired via horizontal gene transfer (Wyres et al., 2020). *K. pneumoniae* is often part of the healthy human microbiota. Recent clinical data indicate that gastrointestinal carriage is a major reservoir of *K. pneumoniae* infections in healthcare environments (Gorrie et al., 2017). A continuous “sensing and responding” to changing physio-chemical conditions occurs in bacteria moving in the gastro-intestinal (GI) tract. Depending on oxygen availability, presence of bile salts, pH and other stress conditions bacteria recognize different gastro-intestinal regions as different microenvironments, adopt diversified strategies to survive in these niches and produce a complex arsenal of virulence factors (Sengupta et al., 2014). The activation of specific efflux pumps is regarded as a major strategy to keep the intracellular concentration of toxic substrates (e.g., bile salts, molecules with antimicrobial activity, oxidative agents) at a sustainable level (Henderson et al., 2021).

Sequencing of *K. pneumoniae* genomes has revealed that genes encoding EPs potentially responsible for extruding antibiotics represent up to 10% of the total gene pool. More than ten EPs belong to the MFS family. At present antibiotic extrusion has been clearly demonstrated only for a few EPs and the physiological relevance of these genes is still under investigation. In this respect interesting data exists on the biological function of KpnGH, a multidrug EP of the MFS family. It consists of two subunits, KpnG and KpnH, which display a high protein and nucleotide sequence similarity with the EmrA adaptor protein and the EmrB transporter of the EmrAB EP of *E. coli* (Srinivasan et al., 2014). KpnGH confers resistance to a large panel of antimicrobial compounds, cationic dyes, antiseptics chemicals and detergents. The contribution of KpnGH to withstand the harsh conditions *K. pneumoniae* encounters in the human GI tract derives from the observation that a *kpnGH* mutant is more sensitive to bile salts, suggesting that *K. pneumoniae* uses this EP to circumvent the inhibitory effect of bile salts. The ability to confer resistance to bile salts is found also in *E. coli* EmrAB and in the closely related *V. cholera* VceCAB (Woolley et al., 2005), suggesting that in several bacterial pathogens EmrAB-like EPs contribute significantly to the survival in bile-rich environments. Further evidence obtained by comparing wt and *kpnGH*-defective *K. pneumoniae* strains confirm that KpnGH favours survival under high osmotic environments and oxidative or nitrosative stress (Srinivasan et al., 2014). Overall, these data suggest an active role of KpnGH in supporting the bacterium under conditions typically found in the upper parts of the GI, characterized by severe osmotic condition in a microaerobic environment.

## Conclusion

The large amount of experimental evidence accumulated over the last years strongly suggests that the function of MDR EPs in many bacterial pathogens is not strictly limited to antibiotic resistance but rather has a wider impact on bacterial interactions with host cells. Studying the interplay between bacteria and hosts is an effective approach to fully understand the physiological significance of MDR EPs. The MFS superfamily constitutes the largest group of transporters and is present in all phyla. The model systems we have presented in this review illustrate the many roles MFS EPs play in the communication between bacteria and host cells, in particular in key phases of a pathogen’s life, as adhesion, invasion, intracellular survival and biofilm formation. The complex regulation of several EPs by global networks, as the TCS system, reflects the need of the bacterial cell to fine tune the expression of MDR EPs in response to specific niches and in coordination with other virulence factors. The possibility to block the communication with target host cells by inhibiting the expression or the activity of MDR EPs is a promising strategy not only to combat the onset of drug resistances but also to attenuate virulence. Several inhibitors targeting EPs or their regulators have been discovered, including natural products, antibiotics and synthetic molecules (Sun et al., 2014; Wang et al., 2016; Eguchi et al., 2017). Very recently a novel strategy based on natural-like compounds able to outcompete antibiotic efflux has been envisioned, opening stimulating perspectives in counteracting EP-mediated drug resistance and virulence in bacterial pathogens (Laborda et al., 2021).

## Abbreviations

ABC, ATP binding cassette; AbgT, p-aminobenzoyl-glutamate transporter; AIEC, adherent invasive *Escherichia coli*; CD, crohn disease; c-di-AMP, cyclic-di-AMP; CR, congo red; DHA, drug/H^+^ antiporter; DMT, drug/metabolite trasporter; EP, efflux pump; GFP, green fluorescence protein; IFN-β, interferon-β; IM, inner membrane; LTA, lipoteichoic acid; MATE, multidrug and toxic compound efflux; MDR, multidrug resistance; MFS, major facilitator superfamily; MTAC, MdrMATC; NF-kB, nuclear factor kB; OM, outer membrane; OMF, outer membrane factor; PACE, proteobacterial antimicrobial compound efflux; PAP, periplasmatic adaptor protein; PG, peptidoglycan; RECON, reductase controlling NF-κB; RND, resistance nodulation division; TCS, two component system; TM, trans membrane; TLR, toll-like receptor; WTA, wall teichoic acid.
